# Lifetime cost-effectiveness analysis of intraoperative radiation therapy versus external beam radiation therapy for early stage breast cancer

**DOI:** 10.1186/s12962-017-0084-5

**Published:** 2017-11-09

**Authors:** Rakesh Patel, Olga Ivanov, Jeff Voigt

**Affiliations:** 1 Radiation Oncology, Good Samaritan Hospital, 425 Samaritan Dr, San Jose, CA 95124 USA; 2 Breast Health Center, Celebration Health, 2nd Floor, 380 Celebration Pl, Celebration, FL 34747 USA; 3 Medical Device Consultants of Ridgewood, LLC, 99 Glenwood Rd., Ridgewood, NJ 07450 USA

**Keywords:** IORT, EBRT, Cost-effectiveness, Breast cancer

## Abstract

**Background:**

To date no one has examined the quality of life and direct costs of care in treating early stage breast cancer with adjunct intraoperative radiation therapy (IORT) versus external beam radiation therapy (EBRT) over the life of the patient. As well no one has examined the effects of radiation exposure with both therapies on the longer term sequelae. The purpose of this analysis was to examine the cost-effectiveness of IORT vs. EBRT over the life of the patient.

**Methods:**

A Markov decision-analytic model evaluated these treatment strategies in terms of the direct costs in treating patients over their lifetime (including the downstream costs associated with radiation exposure) and the resultant quality of life of these patients. Medicare reimbursement amounts in treating patients were used for acute, steady state, recurrent cancer(s), and complications associated with radiation exposure. Quality adjusted life years (QALYs) derived from the medical literature were assessed with each of these states. Life expectancies as well were derived from the medical literature. Cost-effectiveness was evaluated for dominance and net monetary benefit [at a willingness to pay (WTP)] of $50,000/QALY. Sensitivity analysis was also performed.

**Results:**

IORT was the dominant (least costly with greater QALYs) versus EBRT: total costs over the life of the patient = $53,179 (IORT) vs. $63,828 (EBRT) and total QALYs: 17.86 (IORT) vs. 17.06 (EBRT). At a willingness to pay of $50,000 for each additional QALY, the net monetary benefit demonstrated that IORT was the most cost effective option: $839,815 vs. $789,092. The model was most sensitive to the probabilities of recurrent cancer and death for both IORT and EBRT.

**Conclusion:**

IORT is the more valuable (lower cost with improved QALYs) strategy for use in patients presenting with early stage ER+ breast cancer. It should be used preferentially in these patients.

**Electronic supplementary material:**

The online version of this article (10.1186/s12962-017-0084-5) contains supplementary material, which is available to authorized users.

## Background

In the United States, there are approximately, 60,000 new cases yearly of in situ breast cancer where a large portion of these patients may be indicated for adjunctive radiation therapy [1]. Intraoperative radiation therapy (IORT) may be considered an option to external beam radiation therapy (EBRT) as adjunctive therapy post lumpectomy for locoregional treatment of early stage breast cancer due to a clinical dilemma—some patients may be unable to attend the lengthy treatment course of EBRT (up to 6 weeks); some may find it stressful; and some may be looking for a better patient experience [2]. Recent cost effectiveness analysis comparing intraoperative radiation therapy (IORT) to external beam radiation therapy (EBRT) as part of adjunctive therapy for this type of early stage breast cancer has demonstrated that IORT provides greater quality-adjusted life years (QALYs—a summary measure of health outcome used in economic evaluation, and incorporate the impact of the quantity and quality of life) at a decreased direct cost (i.e. a dominant strategy) when compared to a 6 week regimen of whole breast (WB)—EBRT [3]. This analysis however only examined cost-effectiveness over a 10 year timeframe and did not examine the longer term sequelae associated with radiation exposure from both EBRT and IORT [2]. Another study examined patients 12 years out and found similar findings of cost effectiveness (examining direct costs) with IORT [4]. Lastly, a third study by the same author as in [4], examined both direct and indirect costs and found that EBRT was a more cost-effective therapy compared to IORT [5]. We believe, based on an extensive search of the literature, that no one to date has examined the direct costs of treatment and long term sequelae over the life of the patient.

Recent studies have shown an increase in major coronary events (MCEs) (i.e. myocardial infarction, coronary revascularization, or death from ischemic heart disease) based on dosage exposure to radiation [6, 7]. Additionally, the risk of second solid cancers after radiotherapy for breast cancer is also increased [8, 9]. These events not only increase the direct costs in treating these patients over their entire life but as well affect their overall quality of life [10–15].

Based on the above, we examined the direct costs and quality of life over the lifetime of the patients in IORT versus EBRT, taking into account newer data and the potential complications associated with radiation exposure. The viewpoint of this analysis was from the health payer perspective.

## Methods

A Markov decision tree model (TreeAge Pro 2016; Williamstown, MA) using data obtained from the peer reviewed literature compared the use of IORT versus 6 week WB-EBRT in treating early stage (stage I–IIA/IIB) breast cancer. TreeAge Pro 2016 is a widely accepted and used modeling software program used in decision tree and Markov modeling. This course of WB-EBRT is consistent with prior analyses [3]. The model also used the same cohort of patients (55 year old females) studied previously [3]. The assumptions made with radiation mean exposure were 5 gray units (Gy) with EBRT for the heart (which is the average dose the left and right breast) [7]; 3.8 Gy with EBRT for the ipsilateral lung [9]; and 1.1 Gy for the contralateral breast [9]. For IORT, the radiation mean exposures were as follows: 1.25 Gy (heart); 0.03 Gy for ipsilateral lung; and 0 Gy for the contralateral breast [9]. A significant portion of the model’s effectiveness data was derived from a multicenter (33 sites); 11 country, randomized controlled trial, which enrolled over 3300 patients [2, 16]. Based on the size of this study and the fact that it was multicenter, and that it followed patients over 5 years, we believe it was a sufficient source of the clinical effectiveness of IORT compared to EBRT. Further, the literature review process in gaining additional inputs (for health utilities, life expectancies and probabilities, costs) entailed a PubMed search using the following terms: breast cancer AND recur* AND complicat*. A separate PubMed literature search was also undertaken using the following terms: breast cancer AND radiation AND complications. A third PubMed literature search used the following terms: breast cancer AND QoL. Finally, a fourth PubMed literature search was performed using the following terms: breast cancer AND surgery AND radiation therapy AND cost effectiveness. Lastly, the National Cancer Institute (NCI) Surveillance Epidemiology and End Result (SEER) [17] was searched for life expectancy in breast cancer patients where lumpectomy plus radiation were treatments. These searches were undertaken on November 15, 2016 except for the 4th PubMed search which was undertaken on October 14, 2017.

### Model structure

**Fig. 1:**
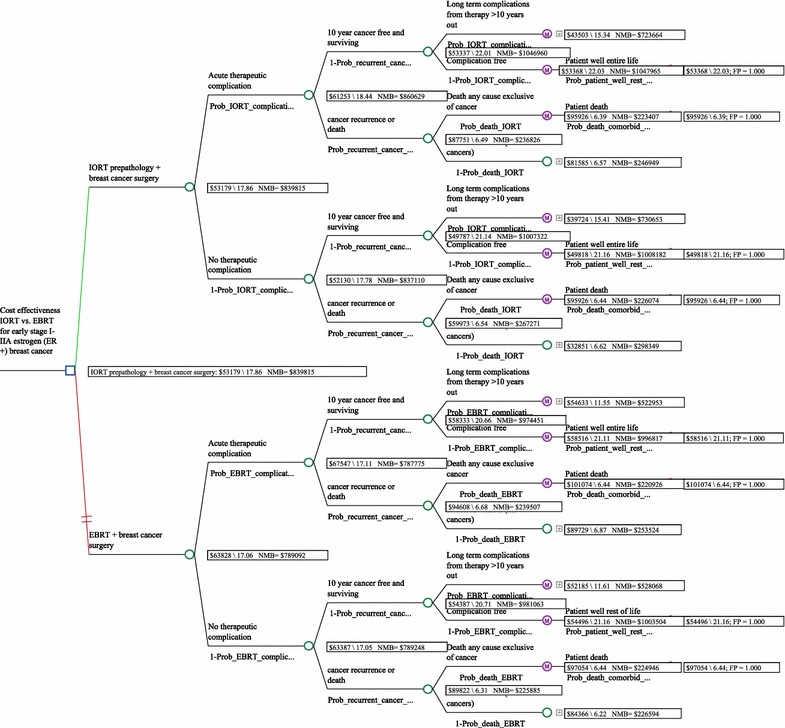
Decision tree

All women were initially treated with breast conserving surgery (BCS) and followed by either IORT or 6 weeks of EBRT [18]. Acute post surgery and radiation complication rates were evaluated based on prior studies [2, 16]. After the initial acute care episode, patients entered into a steady state of health (with associated costs and health utilities; derived from publications). Patients then transitioned from this steady state into other health states over time based on probabilities obtained from the medical literature [e.g. comorbid conditions, recurrent cancers (local, regional, metastatic), complications associated with radiation exposure] and life expectancies based on these conditions. Figure 1 displays the model. Additional file 1: Appendix S1 shows these probabilities, costs, utilities, and life expectancies.

**Fig. 2:**
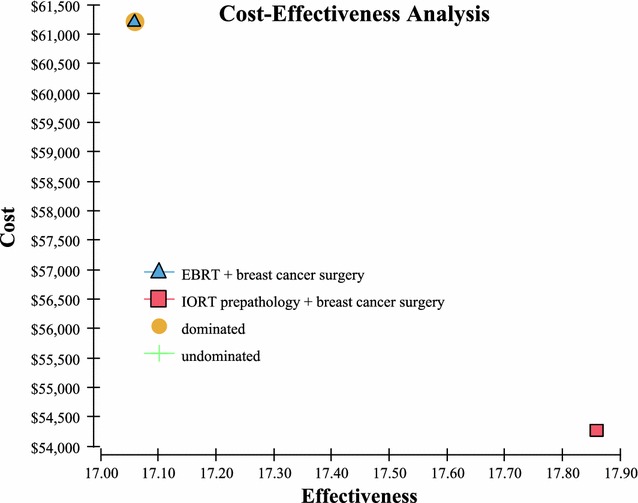
Cost effectiveness graph

### Health utilities

Where possible patient preferences regarding tradeoffs for health utilities and EuroQoL VAS patient reported assessments were used [10, 11, 13, 15, 19, 20]. Tradeoffs are commonly used in assessing patient preferences for one therapy vs. another and a resultant outcome, e.g. breast surgery alone vs. breast surgery plus radiation and the resultant outcome of a local recurrence of breast cancer [21]. These values were varied in sensitivity analysis within clinically reasonable ranges (in other words what would be found in studies where these health utilities were evaluated) and; as well to determine threshold values when one therapy dominated the other on health utility versus another. A discount rate of 3%/year was used for utilities estimated out into the future [22]. These health utilities were aggregated over time and reported on using QALYs.

### Life expectancies and probabilities

Life expectancies for patients with no recurrence and recurrence (local, regional, metastatic breast cancer), comorbid conditions, long term complications due to radiation exposure and were derived from the literature [23–28]. In some instances life expectancies were weighted based on the complication incidences due to radiation exposures as outlined above (Additional file 2: Appendix S2). Probabilities of other events (e.g. acute complications; long term complication associated with radiation exposure; cancer recurrence, death, and comorbid conditions were derived from the literature [2, 7, 9, 16, 25, 29]. Life expectancies were used in the Markov model as cycles (years of remaining life) and are found in Table 1.

**Table 1:** Cycle lengths (life expectancy) used based on patient condition

Patient condition	IORT cycle length (estimated length of life in years)	EBRT cycle length (estimated length of life in years)
Long term complications resulting from procedure after being cancer free for 10 years	16	14
Complication free	23	23
Recurrent local/regional cancer	10	10
Metastatic cancer	6	6
Death from comorbid conditions exhibited by cancer patients	4.2–7.9	4.2–7.9

### Costs

As with the prior cost-effectiveness study [3], direct costs of care for the initial episode of care (BCS plus radiation exposure) and associated complications (including the incidence of) [16] were estimated using medicare reimbursement. With follow on care associated with “well care” (care for a patient who is cancer free and in good health), recurrence of breast cancer and, long term complications associated with radiation exposure, care in the last year of life direct cost estimates—all these were used from published data [30]. Lastly with long term complications, a weighted average of the costs (initial, ongoing and last year of life for MCE, lung cancer and breast cancer) and using associated long term complication incidences was derived from publicly available sources (i.e. electronic data sets such as SEER) [31] and from the peer reviewed literature [32]. This weighting was performed based on an assumed exposure to radiation, the complication rate based on that exposure and a weighting of a complication as a percent of all complications seen from heart disease, lung cancer and contralateral breast cancer (see Additional file 2: Appendix S2). As mentioned above, the incidence of the long term complications was based on the exposure of the patient to the radiation therapy [7, 9, 15] Some of these estimates were inflated to the year 2016 using the medical service CPI based on the year used [33]. A discount rate of 3%/year was used for costs estimated out into the future [21]. As mentioned above the perspective taken with this study is that of the payer of the direct costs for care, e.g. insurers and patients.

### Model outputs

Both IORT and EBRT used as adjunctive therapies to BCS were evaluated for direct medical costs and health utilities over the entire remaining life of the patient. Each strategy was evaluated for dominance—meaning either a lower cost alternative or a higher health utility. Further, each was evaluated for its net monetary benefit (NMB), which is a combination of cost, effectiveness (QALYs) and willingness to pay (WTP). This measure is used to identify the alternative with the highest net benefit as being the most cost-effective given a certain WTP. The WTP used in this model is $50,000/QALYs gained [34]. The rationale for choosing $50,000/QALY gained as elucidated in Neumann et al. [33] in that this amount has been most commonly used in cost effectiveness analysis during the 1990–2012 timeframe. Willingness to pay means that for any given therapy, one is willing to pay that amount for keeping one in perfect health for a given timeframe (typically a year)—in this case $50,000 for a year of perfect health. The NMB of each alternative is calculated using the following formula: NMB = aggregate lifetime health utilities (or QALYs) × WTP less aggregate lifetime costs.

## Results

### Baseline analysis

The Markov model demonstrates that over the life of the patient, IORT is the dominant strategy—it is less costly, provides for higher QALYs, and results in the highest NMB and a lower cost per QALY compared to EBRT: $3039 vs. $3741 respectively (Table 2).

**Table 2:** This dominance is further demonstrated in Fig. 2

Alternative	Direct cost	QALYs	Cost/QALY	NMB
IORT	$53,179	17.86	$2978	$839,815
EBRT	$63,828	17.06	$3741	$789,092

### Sensitivity analysis

**Fig. 3:**
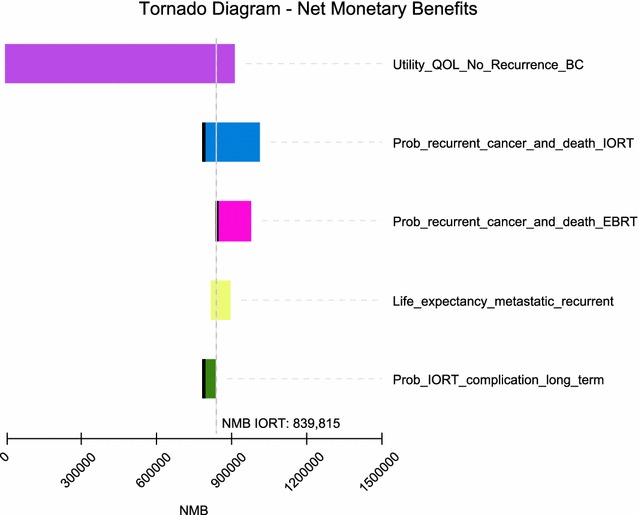
Tornado diagram

Based on findings from the tornado plot which evaluated the NMB of the alternative with the highest net benefit (Fig. 3) and at a willingness to pay (WTP) of $50,000 for each quality adjusted life year (QALY), the Markov model was most sensitive to the following main variables in the model (Table 3).

**Fig. 4:**
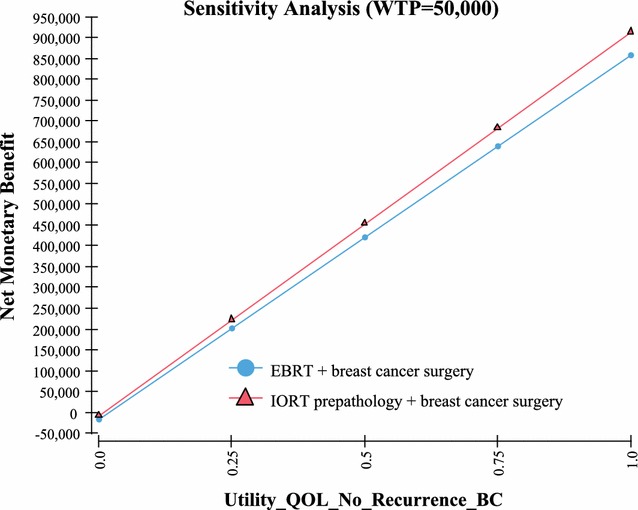
Sensitivity analysis—quality of life no recurrent breast cancer

**Fig. 5:**
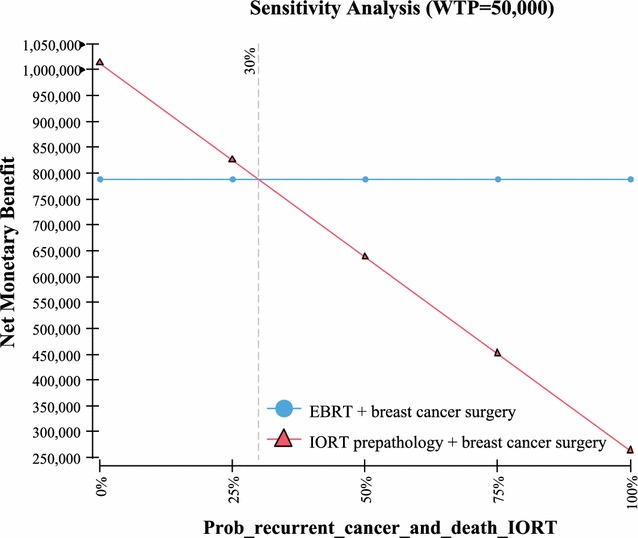
Sensitivity analysis—probability recurrent events IORT

**Fig. 6:**
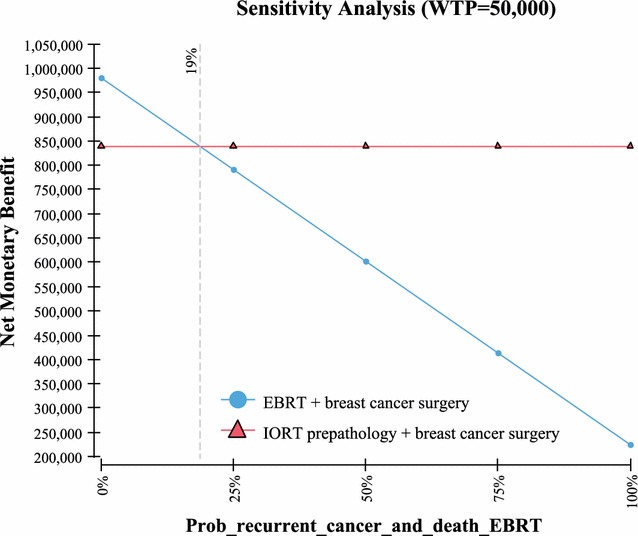
Sensitivity analysis—probability recurrent events EBRT

**Fig. 7:**
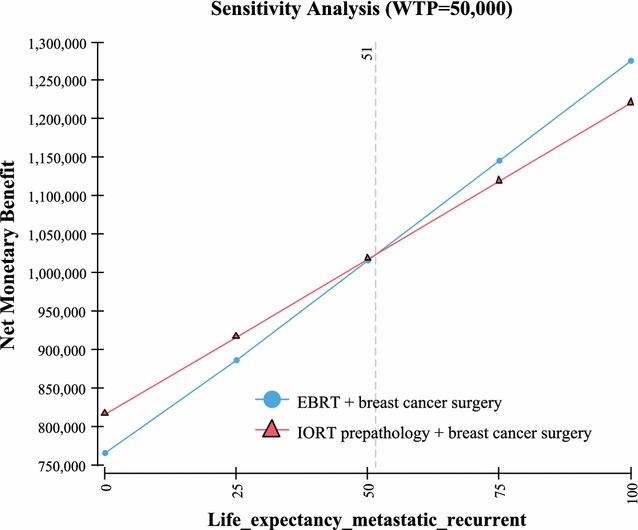
Sensitivity analysis—life expectancy

**Fig. 8:**
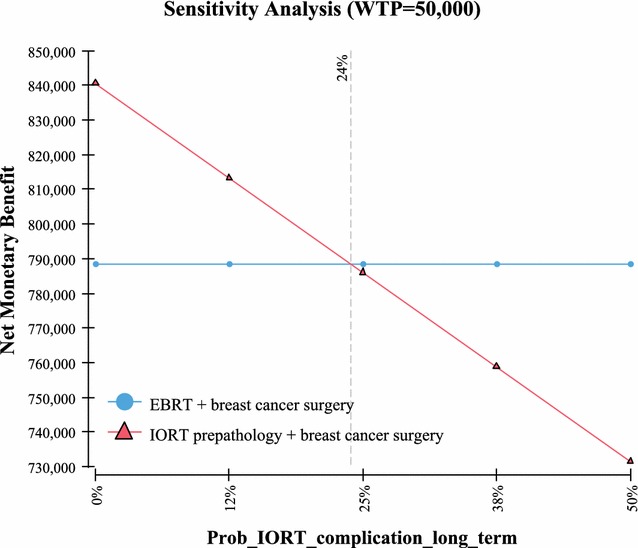
Sensitivity analysis—probability complications (> 10 years) IORT

**Table 3:** One way sensitivity analysis

Variable	Value used in the Markov model	Value at which IORT no longer has the highest NMB
Utility QoL with no recurrence of breast cancer based on a scale of 0–1; with “1” being in perfect health and “0” resulting in death (Fig. 4)	0.92 [19]	0
Probability of recurrent cancer and death IORT (includes local and other cancer and death) > 10 years out (Fig. 5)	23% [2]	> 30%
Probability of recurrent cancer and death EBRT (includes local and other cancer and death) > 10 years out (Fig. 6)	25.4% [2]	< 19%
Life expectancy metastatic recurrent cancer (Fig. 7)	5.625 year and 95% CI of 5.43–5.82 [23, 24]	> 51 years
Probability IORT complication long term (> 10 years) due to radiation exposure. Includes ischemic heart disease and cancers (Fig. 8)	0.31% [7]	> 24%

## Discussion

This analysis is a follow on study to a prior cost effectiveness analysis [3] and builds upon the prior finding that IORT is the more cost-effective option relative to EBRT when treating middle aged females with early stage breast cancer. The important additions which aid in cost-effectiveness decision making is that this analysis provides an examination of the longer term sequelae of radiation exposure over the life of the patient. This is an important consideration for providers and women when evaluating which therapy to use in treating early stage breast cancer. This study shows there is a significant risk of MCE and lung cancer based on a 4 × radiation exposure with EBRT relative to IORT [7]. This increased radiation exposure translates into an over 15 times relative risk of longer term complications with EBRT [7]. This was a major factor in both the increased costs and decreased health utilities seen with EBRT as used in the Markov model. These findings are not surprising considering in follow on analysis to Vaidya 2010 [2], it was demonstrated that while the overall breast cancer mortality was the same, that there were significantly fewer non-breast cancer deaths, attributable to fewer deaths with IORT from cardiovascular causes and other cancers: 1.4%, 95% CI 0.8–2.5% vs. 3.5%, 95% CI 2.3–5.2% with EBRT; P = 0.0086 [2].

This analysis is also important to clinicians due to the fact that the US health care system is moving from a payment system that relied on volume (fee for service) to one that relies on value (determining the most appropriate therapy based on a balancing of clinical outcomes and overall cost to the system, i.e. cost effectiveness). This is based on initiatives as laid out in the passage of the patient protection and affordable care act (PPACA) passed in March of 2010. Cost effectiveness analyses examine what the costs (either incremental or cost savings) are to the system based on unit of effectiveness (some outcome measure—typically that relates to the overall health of the patient, e.g. quality of life). Moving forward, the US health care system’s payments will be tied to clinical outcomes. Therefore an understanding of the concepts behind cost effectiveness by clinicians will enhance their ability to identify which therapies provide the best value for their patients (outcome per dollar spent) while spending healthcare dollars to be spent in a responsible manner.

This analysis is also important relative to the total direct cost savings to the healthcare system. With over 60,000 females diagnosed with in situ breast cancer per year and at a lifetime savings of over $10,500/patient, the use of IORT vs. EBRT in these types of patients represents an overall direct cost savings of $630 million to the US healthcare system.

One of the potential advantages not elaborated on as this analysis, is the potential for patients to recover faster with IORT versus EBRT—considering IORT can be delivered in one session versus 5–6 weeks of EBRT. This in turn may allow patients to return to daily activities faster (including work) [2]. The use of IORT may also include other benefits (over EBRT) such as: costs of travel for patients [2]; and further lost productivity due to the sequelae from EBRT and IORT [5]. If one were to assume that the indirect costs for 6 weeks of treatment were $1520 higher with EBRT (2011 CPI numbers adjusted for inflation to the year 2016) [3], then the additional costs using EBRT would be (60,000 × $1520) $91.2 million in the US.

This paper also suggests that other data be examined in making coverage determinations, including the long term sequelae (e.g. MCE), associated with radiation exposure that have been reported on in peer reviewed journals such as the New England Journal of Medicine [7] and Lancet [2].

The model’s sensitivity to the probabilities of recurrent cancer due to breast cancer and death for both IORT and EBRT is an interesting finding. Based on a 10 year extrapolation of local, recurrent and death events similar to the methodology used in Vaidya 2014 [2] (as per Table 1 for pre-pathology events in the Vaidya 2014 study), it was found that the cumulative probability of events for local, recurrent, and death were similar between IORT (23%) vs EBRT (25.4%). Using these values in the Markov model demonstrated in sensitivity analysis that in order for EBRT to have a higher NMB than IORT (in other words most cost-effective), then the EBRT probability have to be < 19%. Since the probability at 5 years is reported at 12.7% [2], and local, other and death would not be expected to be 1–2%/year (considering it has been growing at ~ 2.5%/year) for the following 5 years, this < 19% probability would be highly unlikely to occur. Further, since the probability of these events occurring in IORT is estimated to be 23% at 10 years, again sensitivity analysis in the Markov model demonstrated that in order for EBRT to have a higher NMB than IORT, the probability of these events would need to be > 30% in IORT patients. Since this would be 25+% higher than the expected 23% (0.29/0.23 = 1.26), again this would be unlikely to occur. The other variables examined, the QoL utilities in patients who are deemed healthy (i.e. no recurrent breast cancer) demonstrated that in order for EBRT to be comparable in NMB to IORT, the QoL value would need to be “0”. In other words the patient would be in very poor health. Since studies have demonstrated that the QoL is in the 0.9 range [19], again this is a value unlikely to occur in real life. Lastly, it was found that in order for ERBT result in a higher NMB benefit, a patient would need to live in excess of 50 years with a diagnosis of metastatic recurrent breast cancer. Again since the estimate of years lived post diagnosis of metastatic breast cancer is 5.6 years [23, 24], this would be an event unlikely to occur.

As mentioned, two prior analyses of the longer term (10 and 2 year) direct costs of IORT vs. EBRT identified cost savings with EBRT [3, 4]. A second analysis however did not [5]. The differences between these analyses and Shah et al. [5], was that the Shah analysis [5] examined direct and indirect (nonmedical) costs whereas this analysis only examined direct costs. Further, the Shah analysis [5] incorporated the increased medical costs associated with operative time with IORT whereas this current analysis did not and used 2016 national average medicare reimbursement as a proxy for overall costs. It is interesting to note that medicare reimbursement is closely tied to costs [35].

Study limitations include a lack of longer term data due to a lack of it being available and the need to extrapolate it using other available reports. An important contribution of Markov modeling is exactly that, to extrapolate near term findings to the longer term using stochastic processes. While Markov model findings are not predictive precisely, they allow for statistical analysis that may guide future prospective studies in this area. Further, longer term studies with expensive therapies such as IORT vs. EBRT are likely cost prohibitive. Thus, findings using Markov modeling may also guide future research via identifying relevant variables to evaluate which may in turn make future research less costly. It is noted however, this need to extrapolate data to use in the model (e.g. 10 year extrapolation of 5 year IORT and EBRT local, recurrent and death events as found in Vaidya 2014 [2]) is a limitation of this analysis and may reduce the face validity of the findings. As well, another limitation is medicare reimbursement data being used for costs. medicare reimbursement data (for hospital care) has been shown to be approximately 94% of total cost—thus the cost estimates contained herein may actually be under-representing the true costs [34]. Thirdly, this current analysis used an average radiation dose (for left and right breast) of 5 Gy. It should also be noted that radiation exposure with ERBT even to the right breast, still resulted in a 20–25% increase in the MCE rate versus IORT [7]. Fourth, the input parameters for a large portion of this analysis were obtained from single clinical study (e.g. probabilities of transition health states). It has been noted that using a single study only can cause high risk of bias that lead to reliability of results [36]. However, the clinical study which was most referenced for probabilities was a multicenter (33 centers), 11 country, with close to 3500 randomized controlled patient evaluation of IORT vs. EBRT [2]. We believe that such a large study minimizes these biases.

Lastly, this analysis was undertaken on middle aged women expected to live at least 23 ± 4 years (without recurrence) after their initial episode of early stage breast cancer.

## Conclusions

In summary, the use of IORT appears to be a dominant strategy and thus should be preferred over whole breast EBRT. In this new era of value based medicine, and based on this analysis, these types of technologies have the potential to save the healthcare system money, while simultaneously providing improved patient outcomes.

## Additional files

**Additional file 1: Appendix S1.** Variables included in the Markov model.

**Additional file 2: Appendix S2.** Long term complications associated with exposure to radiation—life expectancy.

## Abbreviations

BCS: breast conserving surgery
EBRT: external bean radiation therapy
ER+: estrogen receptor positive
Gy: gray units
IORT: intraoperative radiation therapy
MCE: major coronary events
NMB: net monetary benefit
PPACA: patient protection and affordable care act
QALYs: quality adjusted life years
WB: whole breast
WTP: willingness to pay
