# Supplementary material for: Lifetime cost-effectiveness analysis of intraoperative radiation therapy versus external beam radiation therapy for early stage breast cancer
Source: Cost Eff Resour Alloc. 2017 Nov 9;15:22. doi: 10.1186/s12962-017-0084-5 (PMC5679386; doi:10.1186/s12962-017-0084-5)
Supplement: Supplementary file 1 — Additional file 1: Appendix S1. Variables included in the Markov model. [file 12962_2017_84_MOESM1_ESM.docx]

| Appendix S1 Variables included in the Markov model | |  |  |  |  |
| --- | --- | --- | --- | --- | --- |
| **Name** | **Description** | **Comment** | **Root Definition** | **25% less** | **25% higher** |
| Prob_IORT_complication_acute | Probability of an acute IORT complication | Vaidya JS et al. Targeted intraoperative radiotherapy vs whole breast radiotherapy for breast cancer (TARGIT-A). Lancet. 376:91-102. | 0.115 | 0.08625 | 0.14375 |
| Prob_EBRT_complicaton_acute | Probability of an acute complication from EBRT | Vaidya JS et al. Targeted intraoperative radiotherapy vs whole breast radiotherapy for breast cancer (TARGIT-A). Lancet. 376:91-102. | 0.106 | 0.0795 | 0.1325 |
| Prob_recurrent_cancer_and_death_EBRT | Probability of a recurrent cancer (local or other) and death EBRT | Vaidya JS et al. Risk-adapted targeted intraoperative radiotherapy versus whole-breast radiotherapy for breast cancers: 5-year results for local control and overall survival from the TARGIT-A randomized trial. Lancet 2014. 383:601-613. Extrapolation of 5 year events to 10-yr probabilities, using per year transition probabilities for years 6-10 of 0.22% local; 0.94% other; and 1.38% for death. Totals up to 10 year events of: 2.2% local; 9.4% other; and 13.8% death. In total 25.4% | 0.254 | 0 | 0.5 |
| Prob_recurrent_cancer_and_death_IORT | Probability recurrent cancer (local or other) and death IORT | Vaidya JS et al. Risk-adapted targeted intraoperative radiotherapy versus whole-breast radiotherapy for breast cancers: 5-year results for local control and overall survival from the TARGIT-A randomized trial. Lancet 2014. 383:601-613. Extrapolation of 5 year events to 10-yr probabilities, using per year transition probabilities for years 6-10 of 0.42% local; 0.96% other; and 0.92% for death. Totals up to 10 year events of: 4.2% local; 9.6% other; and 9.2% death. In total 23% | 0.23 | 0 | 0.4 |
| Prob_death_EBRT | Probability of death with EBRT arm as compared with other cancer | Derived from: Schairer C, et al. Probabilities of death from breast cancer and other causes among female breast cancer patients. JNCI 2004. 96(17):1311-1321. Other non-cancer causes of death equal 43% in females 50-59 years of age with ER+; <2cm breast cancer | 0.43 | 0.3225 | 0.5375 |
| Prob_death_IORT | Probability of death with IORT as compared to other cancer | Derived from: Schairer C, et al. Probabilities of death from breast cancer and other causes among female breast cancer patients. JNCI 2004. 96(17):1311-1321. Other non-cancer causes of death equal 43% in females 50-59 years of age with ER+; <2cm breast cancer | 0.43 | 0.3225 | 0.5375 |
| Prob_local_cancer_EBRT | Probability of local cancer compared to other cancers EBRT | Vaidya JS et al. Risk-adapted targeted intraoperative radiotherapy versus whole-breast radiotherapy for breast cancers: 5-year results for local control and overall survival from the TARGIT-A randomized trial. Lancet 2014. 383:601-613. Includes: 2.2% local; 9.4% other; and 13.8% death (local cancer [2.2]/total cancer [11.6] = 19%) | 0.19 | 0.1425 | 0.2375 |
| Prob_local_cancer_IORT | Probability local cancer compared to other cancers IORT | Vaidya JS et al. Risk-adapted targeted intraoperative radiotherapy versus whole-breast radiotherapy for breast cancers: 5-year results for local control and overall survival from the TARGIT-A randomized trial. Lancet 2014. 383:601-613. Includes: 4.2% local; 9.6% other; and 9.2% death (4.2/13.8 = 30%) | 0.30 | 0.225 | 0.375 |
| Utility_QOL_Death | Quality of life death |  | 0.0 | 0 | 0 |
| Utility_QOL_No_Recurrence_BC | Health utility per year without breast cancer recurrence | Derived from: Hayman JA, et al. Patient preferences concerning the trade-off between the risks and benefits of routine radiation therapy after conservative surgery for early-stage breast cancer. Jrl Clin Oncol 1997. 15:1252-1260. | 0.92 | 0.69 | 1 |
| Cost_lump_plus_IORT | Cost of treating a patient with surgical lumpectomy plus IORT | Derived from 2016 Medicare reimbursements for: partial mastectomy ($1,767) plus IORT $7,933) plus observation ($210) = $9,910 | $10,248 | $7,686 | $12,810 |
| Cost_lump_plus_6wkEBRT | Cost for surgical lumpectomy plus 6 weeks of EBRT | Derived from 2016 Medicare reimbursements: Surgical mastectomy ($1,977) plus EBRT planning ($3,285) plus ($5,958) = $11,220 | $11,220 | $8,415 | $14,025 |
| Cost_metastatic_BC_last_year | Costs for treating metastatic breast cancer in the last year of life | Source: Yabroff KR, et al. Cost of care for elderly cancer patients in the US. JNCI 2008. 100(8):630-641. Costs were inflated from 2004 to present using Medical Service CPI obtained from the Bureau Labor Statistics | $64,549 | $48,412 | $80,686 |
| Cost_annual_care_ongoing | Cost of annual ongoing care for breast cancer between initial and last 12 months of life | Source: Yabroff KR, et al. Cost of care for elderly cancer patients in the US. JNCI 2008. 100(8):630-641. Costs were inflated from 2004 to present using Medical Service CPI obtained from the Bureau Labor Statistics. Ongoing $1,768 | $1,768 | $1,326 | $2,210 |
| Prob_EBRT_complication_long_term | Probability of a long term complication from EBRT therapy >10 years out | Derived from: Darby SC et al. Risk of ischemic heart disease in women after radiotherapy for breast cancer. NEJM. 2013;368-987-98 (1.6% heart disease); Aziz MH et al. Can the risk of secondary cancer induction after breast conserving therapy be reduced using IORT with low-energy X-rays? Radiation Oncol 2011. 6:174. Ng J, et al. Predicting the risk of secondary lung malignancies associated with whole-breast radiation therapy. Int Jr. Rad Oncol 2011. (2.9% lung cancer; 0.22% contralateral breast cancer). In total 4.72% (Also see Appendix 2) | 0.0472 | 0.0354 | 0.059 |
| Prob_IORT_complication_long_term | Probability of a long term complication due to IORT therapy >10 years out | Derived from: Darby SC et al. Risk of ischemic heart disease in women after radiotherapy for breast cancer. NEJM. 2013;368-987-98 (0.225% heart disease; based on 1.25 GY exposure); Aziz MH et al. Can the risk of secondary cancer induction after breast conserving therapy be reduced using IORT with low-energy X-rays? Radiation Oncol 2011. 6:174. Ng J, et al. Predicting the risk of secondary lung malignancies associated with whole-breast radiation therapy. Int Jr. Rad Oncol 2011. (0.02% lung cancer; 0.22% contralateral breast cancer - based on 0.03GY exposure). In total 0.31% (Also see Appendix 2) | 0.0031 | 0.002325 | 0.003875 |
| Cost_acute_complications_IORT | Cost of acute complications associated with adjuvant IORT therapy for stage 1 or 2 BC | Derived from: Vaidya JS, et al. Lancet 2010. 376:91-102. Includes list of complications from table 5 of study which have been monetized using 2016 Medicare reimbursement rates | $3,550 | $2,663 | $4,438 |
| Cost_acute_complications_EBRT | Cost of acute complications associated with adjuvant EBRT therapy for stage 1 or 2 BC | Derived from: Vaidya JS, et al. Lancet 2010. 376:91-102. Includes list of complications from table 5 of study which have been monetized using 2016 Medicare reimbursement rates | $4,020 | $3,015 | $5,025 |
| LE_long_term_comps_after_comp_EBRT | Life expectancy after a EBRT associated long term complication | Life expectancy of a patient who experiences a long term complication as a result of treatment from radiation exposure due to EBRT. Long term complications include: CVD, lung, and breast cancer. (Also see Appendix 2) | 2.51 | 1.8825 | 3.1375 |
| LE_long_term_comps_after_comp_IORT | Life expectancy after a IORT associated long term complication | Life expectancy of a patient who experiences a long term complication as a result of treatment from radiation exposure due to IORT. Long term complications include: CVD, lung, and breast cancer (Also see Appendix 2) | 4.18 | 3.135 | 5.225 |
| Utility_LT_comps_radiation_initial_EBRT | Health utility from LT complications associated with radiation exposure initial - EBRT | Weighted and combined health utility associated with the complications associated with radiation of a patient who would have CVD or lung cancer or recurrent BC - based on health utilities at the start of treatment (Also see Appendix 2) | 0.70 | 0.525 | 0.875 |
| Utility_LT_comps_radiation_initial_IORT | Health utility from LT complications associated with radiation exposure initial - IORT | Weighted and combined health utility associated with the complications associated with radiation of a patient who would have CVD or lung cancer or recurrent BC - based on health utilities at the start of treatment (Also see Appendix 2) | 0.79 | 0.5925 | 0.9875 |
| Utility_LT_comps_radiation_steady_state_EBRT | Health utility from LT complications associated with radiation exposure steady state - EBRT | Weighted and combined health utility associated with the complications associated with radiation of a patient who would have CVD or lung cancer or recurrent BC - steady state condition (Also see Appendix 2) | 0.73 | 0.5475 | 0.9125 |
| Utility_LT_comps_radiation_steady_state_IORT | Health utility from LT complications associated with radiation exposure steady state - IORT | Weighted and combined health utility associated with the complications associated with radiation of a patient who would have CVD or lung cancer or recurrent BC - steady state condition (Also see Appendix 2) | 0.77 | 0.5775 | 0.9625 |
| Cost_LT_comps_initial_EBRT | Cost in treating a long term complication resulting from exposure to radiation initial - EBRT | Weighted and combined health costs associated with the complications associated with radiation of a patient who would have CVD or lung cancer or recurrent BC - initial treatment of complication (Also see Appendix 2) | $41,436 | $31,077 | $51,795 |
| Cost_LT_comps_initial_IORT | Cost in treating a long term complication resulting from exposure to radiation initial - IORT | Weighted and combined health costs associated with the complications associated with radiation of a patient who would have CVD or lung cancer or recurrent BC - initial treatment of complication (Also see Appendix 2) | $25,865 | $19,399 | $32,331 |
| Cost_LT_comps_steady_state_EBRT | Cost in treating a long term complication resulting from exposure to radiation steady state - EBRT | Weighted and combined health costs associated with the complications associated with radiation of a patient who would have CVD or lung cancer or recurrent BC - steady state treatment of complication (Also see Appendix 2) | $11,545 | $8,659 | $14,431 |
| Cost_LT_comps_steady_state_IORT | Cost in treating a long term complication resulting from exposure to radiation steady state - IORT | Weighted and combined health costs associated with the complications associated with radiation of a patient who would have CVD or lung cancer or recurrent BC - steady state treatment of complication (Also see Appendix 2) | $18,066 | $13,550 | $22,583 |
| Cost_LT_comps_end_of_life_EBRT | Cost in treating a long term complication resulting from exposure to radiation endo of life - EBRT | Weighted and combined health costs associated with the complications associated with radiation of a patient who would have CVD or lung cancer or recurrent BC - end of life treatment of complication (Also see Appendix 2) | $67,304 | $50,478 | $84,130 |
| Cost_LT_comps_end_of_life_IORT | Cost in treating a long term complication resulting from exposure to radiation endo of life - IORT | Weighted and combined health costs associated with the complications associated with radiation of a patient who would have CVD or lung cancer or recurrent BC - end of life treatment of complication (Also see Appendix 2) | $55,046 | $41,285 | $68,808 |
| LE_prior_to_LT_complication_IORT | Life expectancy BC patient who is cancer free for >10 years prior to long term complication due to IORT | Weighted average of time in years a patient who is cancer free for >10 years after IORT treatment would live without a long term complication resulting from IORT therapy (CVD, lung cancer, breast cancer) (Also see Appendix 2) | 11.68 | 8.76 | 14.6 |
| LE_prior_to_LT_complication_EBRT | Life expectancy BC patient who is cancer free for >10 years prior to long term complication due to EBRT | Life expectancy of a patient who experiences a long term complication as a result of treatment from radiation exposure due to EBRT. Long term complications include: CVD, lung, and breast cancer (Also see Appendix 2) | 11.01 | 8.2575 | 13.7625 |
| Cost_treat_other_causes_last_yr_life | Cost to treat other causes in the last year of life | Derived from: Levinsky NG, et al. Influence of age on Medicare expenditures and medical care in the last year of life. JAMA. 2001. 286:1349-1355. 1996 $ inflated to 2016 using medical service CPI | $72,614 | $54,461 | $90,768 |
| Life_expectancy_no_recurrence | Life expectancy with no recurrence of BC | Derived from: Capocaccia R, et al. Life expectancy of colon,  breast, and testicular cancer patients: an analysis of US-SEER  population-based data. Annals Oncol 2015. 26:1263-1268. Analysis takes into account no recurrence of cancer | 22.3 | 16.725 | 27.875 |
| Life_expectancy_metastatic_recurrent | Life expectancy with metastatic breast cancer | Touboul E, et al. Local recurrences and distant metastases after breast-conserving surgery and radiation therapy for early breast cancer. Int Jr. Rad Oncol 1999. 43(1):25-38. Chang J, et al. Survival of patients with metastatic breast carcinoma. Cancer 2003;97:545-553. Survival in years in patients with ER+ metastatic recurrent breast cancer. Table 2; page 549.  Combining means and 95% CI results in a mean of 5.625 year and 95% CI of 5.43 to 5.82. (see Appendix 2 for detailed calculations) | 5.625 | 4.21875 | 7.03125 |
| Life_expectancy_locoregional_recurrent | Life expectancy with a local or regional recurrent breast cancer | Touboul E, et al. Local recurrences and distant metastases after breast-conserving surgery and radiation therapy for early breast cancer. Int Jr. Rad Oncol 1999. 43(1):25-38. Kim H, et el. Prognostic factors for survivals for first relapse in breast cancer patients: analysis of deceased patients. Combining years: time initial to LRR; 5 years +/- 0.5 years and LRR to death 4.31 +/- 2.4 years = 9.31 years 95% CI: 9.09 to 9.53 years (see Appendix 2 for more detailed calculations) | 9.31 | 6.9825 | 11.6375 |
| Life_expectancy_comorbid_condition | Life expectancy with a comorbid condition | Derived from deaths from other causes, Table 2, Schairer C, et al. Probabilities of death from breast cancer and other causes among breast cancer patients. JNCI. 2004. 96(17):1311-1321. Main causes death include: heart disease (22%); circulatory diseases (6%); respiratory diseases (8%); other diseases (32%). Life expectancy with heart disease 50-65 year old is 4.2 - 7.9 years. Derived from: Crimmins EM et al. Life with and without heart disease among women and men. Jrl Women Aging. 2008. 20(1-2):5-19. | 6 | 4.5 | 7.5 |
| Utility_acute_complication | Health utility with an acute complication | Derived from: Hillner BE et al. Efficacy and cost-effectiveness of adjuvant chemotherapy in women with node-negative breast cancer. NEJM. 1991. 324:160-168. | 0.9 | 0.675 | 1 |
| Utility_QOL_initial_knowledge_BC | Health utility upon the initial knowledge of knowing one has breast cancer | Derived from Alvarado MD et al. Cost-effectiveness analysis of intraoperative radiation therapy for early stage breast cancer. Ann Surg Oncol. 2013. 20:2873-2880 | 0.92 | 0.69 | 1 |
| Cost_locoregional_BC_last_year_life | Cost of local or regional breast cancer in the last year of life | Source: Yabroff KR, et al. Cost of care for elderly cancer  patients in the US. JNCI 2008. 100(8):630-641. Costs were  inflated from 2004 to present using Medical Service CPI  obtained from the Bureau Labor Statistics. Local $38,879;  Regional $46,020. Average of the two = $42,050 | $42,450 | $31,838 | $53,063 |

**Distributions used in model**

| \| **NAME** \| **DESCRIPTION** \| **TYPE** \| **PARAMETERS** \| **EV** \| **COMMENT** \| \| --- \| --- \| --- \| --- \| --- \| --- \| \| Cost_local_regional_BC_last_year_life \| Cost treating local or regional BC in the last year of life \| Uniform \| subtype: 2, low: $38,879, high: $46,020 \| 42449.5 \| Source: Yabroff KR, et al. Cost of care for elderly cancer patients in the US. JNCI 2008. 100(8):630-641. Costs were inflated from 2004 to present using Medical Service CPI obtained from the Bureau Labor Statistics. Local $38,879; Regional $46,020. \| \| QOL_initial_knowledge_BC \| QOL patient with the initial knowledge of having breast cancer \| Triangular \| min: 0.87, likeliest: 0.92, max: 0.97 \| 0.92 \| Derived from Alvarado MD et al. Cost-effectiveness analysis of intraoperative radiation therapy for early stage breast cancer. Ann Surg Oncol. 2013. 20:2873-2880 \| \| Life_expect_comorbid_conditions \| Life expectancy of patient with a significant comorbid condition such as heart disease \| Uniform \| subtype: 2, low: 4.2, high: 7.9 \| 6.05 \| Derived from deaths from other causes, Table 2, Schairer C, et al. Probabilities of death from breast cancer and other causes among breast cancer patients. JNCI. 2004. 96(17):1311-1321. Main causes death include: heart disease (22%); circulatory diseases (6%); respiratory diseases (8%); other diseases (32%). Life expectancy with heart disease 50-65 year old is 4.2 - 7.9 years. Derived from: Crimmins EM et al. Life with and without heart disease among women and men. Jrl Women Aging. 2008. 20(1-2):5-19. Crimmins EM et al. Life with and without disease: women experience more of both. 2002. 14(102):47-59. \| \| Breast_Cancer_LE_metastatic_recurrent \| Breast cancer life expectancy metastatic disease \| Triangular \| min: 5.43, likeliest: 5.625, max: 5.82 \| 5.625 \| Touboul E, et al. Local recurrences and distant metastases after breast-conserving surgery and radiation therapy for early breast cancer. Int Jr. Rad Oncol 1999. 43(1):25-38 Chang J, et al. Survival of patients with metastatic breast carcinoma. Cancer 2003;97:545-553. Survival in years in patients with ER+ metastatic recurrent breast cancer. Table 2; page 549. Combining means and 95% CI results in a mean of 5.625 year and 95% CI of 5.43 to 5.82. (Also see Appendix 2). \| \| Breast_Cancer_LE_LR_recurrent \| Breast cancer life expectancy with local regional recurrent \| Triangular \| min: 9.09, likeliest: 9.31, max: 9.53 \| 9.31 \| Touboul E, et al. Local recurrences and distant metastases after breast-conserving surgery and radiation therapy for early breast cancer. Int Jr. Rad Oncol 1999. 43(1):25-38. Kim H, et el. Prognostic factors for survivals for first relapse in breast cancer patients: analysis of deceased patients. Combining years: time initial to LRR; 5 years +/- 0.5 years and LRR to death 4.31 +/- 2.4 years = 9.31 years 95% CI: 9.09 to 9.53 years (Also see Appendix 2 for further calculations) \| \| Breast_Cancer_LE_initial_no_recurrence \| Breast cancer life expectancy initial \| Normal \| mean: 22.3, std dev: 4.6 \| 22.3 \| Derived from: Capocaccia R, et al. Life expectancy of colon, breast, and testicular cancer patients: an analysis of US-SEER population-based data. Annals Oncol 2015. 26:1263-1268. Analysis takes into account no recurrence of cancer. \| \| Complication_acute_health_utility \| Health utility based on acute complication from procedure \| Triangular \| min: 0.7, likeliest: 0.9, max: 1 \| 0.86667 \| Derived from: Hillner BE et al. Efficacy and cost-effectiveness of adjuvant chemotherapy in women women with node-negative breast cancer. NEJM. 1991. 324:160-168. \| \| QOL_first_recurrence_breast_cancer \| Quality of life for a patient with recurrent breast cancer - initial knowledge of. \| Triangular \| min: 0.6, likeliest: 0.7, max: 0.8 \| 0.7 \| Derived from Hillner BE et al. Efficacy and cost effectiveness of adjuvant chemotherapy in women with node-negative breast cancer. NEJM. 1991. 324(30:160-168. \| |
| --- | --- | --- | --- | --- | --- | --- | --- | --- | --- | --- | --- | --- | --- | --- | --- | --- | --- | --- | --- | --- | --- | --- | --- | --- | --- | --- | --- | --- | --- | --- | --- | --- | --- | --- | --- | --- | --- | --- | --- | --- | --- | --- | --- | --- | --- | --- | --- | --- | --- | --- | --- | --- | --- | --- |
|  |
|  |
|  |
|  |
|  |
|  |
|  |
|  |
|  |
|  |
|  |
|  |
