# Supplementary material for: Lifetime cost-effectiveness analysis of intraoperative radiation therapy versus external beam radiation therapy for early stage breast cancer
Source: Cost Eff Resour Alloc. 2017 Nov 9;15:22. doi: 10.1186/s12962-017-0084-5 (PMC5679386; doi:10.1186/s12962-017-0084-5)
Supplement: Supplementary file 2 — Additional file 2: Appendix S2. Long term complications associated with exposure to radiation—life expectancy. [file 12962_2017_84_MOESM2_ESM.docx]

Appendix S2: Long term complications associated with exposure to radiation – life expectancy

|  |  |  | **Comp rate** | **Comp rate** | **Comp as a percent** | **Comp as a percent** |  |  |  |  |
| --- | --- | --- | --- | --- | --- | --- | --- | --- | --- | --- |
| **Complication** | **Therapy** | **Assumed radiation exp** | **IORT** | **EBRT** | **all comps IORT** | **all comps EBRT** | **LE (years)** | **Blended LE EBRT** | **Blended LE IORT** | **Source** |
| Major coronary event [MCE] (MI, death, coronary revascularization) | EBRT | 5 GY; assumes 35% relative increase in rate of MCE; initial rate of with no pre-exist factors 4.5%. 35% increase from 4.5% is 6.1%; absolute increase of 1.6% |  | 1.600% |  | 33.898% | 4.40 | 1.49 |  | Crimmins EM et al. Life with and without heart disease among women and men. Jrl Women Aging. 2008. 20(1-2):5-19. Crimmins EM et al. Life with and without disease: women experience more of both. 2002. 14(102):47-59. |
| Major coronary event [MCE] (MI, death, coronary revascularization) | IORT | 1.25 GY; assumes 5% relative increase in rate of MCE; initial rate of with no pre-exist factors 4.5%. 5% increase from 4.5% is 4.725%; absolute increase of 0.225% | 0.225% |  | 73.770% |  | 4.40 |  | 3.25 | Crimmins EM et al. Life with and without heart disease among women and men. Jrl Women Aging. 2008. 20(1-2):5-19. Crimmins EM et al. Life with and without disease: women experience more of both. 2002. 14(102):47-59. |
| Lung cancer | EBRT | 3.4 GY for ipsilateral lung |  | 2.90% |  | 61.44% | 1.33 | 0.82 |  | Hurria A et al. Management of lung cancer in older adults. CA Cancer J Clin. 2003. 53:325-341 |
| Lung cancer | IORT | 0.03 GY for ipsilateral lung | 0.02% |  | 6.56% |  | 1.33 |  | 0.09 | Hurria A et al. Management of lung cancer in older adults. CA Cancer J Clin. 2003. 53:325-341 |
| Contralateral breast cancer | EBRT |  |  | 0.22% |  | 4.66% | 4.31 | 0.20 |  | Kim H, et el. Prognostic factors for survivals for first relapse in breast cancer patients: analysis  of deceased patients. LRR to death 4.31 +/- 2.4 years |
| Contralateral breast cancer | IORT |  | 0.06% |  | 19.67% |  | 4.31 |  | 0.85 | Kim H, et el. Prognostic factors for survivals for first relapse in breast cancer patients: analysis  of deceased patients. LRR to death 4.31 +/- 2.4 years |
| Total percentages |  |  | 0.31% | 4.72% |  |  |  | 2.51 | 4.18 |  |

Acute care costs of treatment with IORT and EBRT:

| **IORT** | | | |  |  |  |  |  |  |
| --- | --- | --- | --- | --- | --- | --- | --- | --- | --- |
| Medicare 2016 payment rates | | |  |  |  |  |  |  |  |
|  | | |  |  |  |  |  |  |  |
| Source: |  | PFS 2016 and 2016 APC payment rates |  |  |  |  |  |  | **Total** |
| **CPT code** |  | **Description** | **Reimbursement** | **APC** |  | **Description** | **Reimbursement** |  | **Reimburse** |
| **IORT** |  |  |  |  |  |  |  |  |  |
| 19301 |  | Mastectomy, partial (eg lumpectomy, tylectomy, quadrantectomy, segmentectomy) | $673 | 5091 |  | Mastectomy, partial (eg lumpectomy, tylectomy, quadrantectomy, segmentectomy); note: included in 5093 | $0 |  | $673 |
| 19298 |  | Placement radiotherapy afterloading brachytherapy catheter into breast at the time of partial mastectcomy | $338 | 5093 |  | Intraoperative radiation mgmt treatment | $7,558 |  | $7,896 |
| 77469 |  | Intraoperative radiation mgmt treatment | $375 | 5093 |  | Intraoperative radiation mgmt treatment | $0 |  | $375 |
| 99219 |  | Initial observation care | $137 |  |  |  |  |  | $137 |
| 99225 |  | Subsequent observation care | $73 |  |  |  |  |  | $73 |
|  |  |  |  |  |  |  |  |  |  |
| Total payment for outpatient lumpectomy plus IORT | | |  |  |  |  |  |  | $9,154 |

**EBRT acute care treatment costs:**

| **EBRT** |  | **Description** | **Reimbursement** | **APC** |  | **Description** | **Reimbursement** |  | **Total Reim** |
| --- | --- | --- | --- | --- | --- | --- | --- | --- | --- |
| 19301 |  | Mastectomy, partial (eg lumpectomy, tylectomy, quadrantectomy, segmentectomy) | $673 | 5091 |  | Mastectomy, partial (eg lumpectomy, tylectomy, quadrantectomy, segmentectomy) | $2,188 |  | $1,767 |
| 99219 |  | Initial observation care | $137 |  |  |  |  |  | $137 |
| 99225 |  | Subsequent observation care | $73 |  |  |  |  |  | $73 |
|  |  |  |  |  |  |  |  |  |  |
| Total |  |  |  |  |  |  |  |  | $1,977 |
|  |  |  |  |  |  |  |  |  |  |
| **EBRT planning** | | |  |  |  |  |  |  |  |
| 76641 |  | Ultrasound breast, unilateral, complete | $108 | 5531 |  | Ultrasound breast, unilateral, complete | $92 |  | $154 |
| 77014 |  | CT guidance for placement of radiation therapy fields | $119 | N/A |  | CT guidance for placement of radiation therapy fields |  |  | $119 |
| 77263 |  | Therapeutic radiology treatment planning complex | $168 | 5611 |  | Therapeutic radiology treatment planning complex | $107 |  | $222 |
| 77290 |  | complex simulation | $521 | 5613 |  | complex simulation | $292 |  | $667 |
| 77295 |  | 3-D treatment planning | $496 | 5614 |  | 3-D treatment planning | $1,027 |  | $1,523 |
| 77317 |  | brachytherapy isodose planning | $248 | 5613 |  | brachytherapy isodose planning | $292 |  | $394 |
| 77370 |  | special medical radiation physics consult | $123 | 5612 |  | special medical radiation physics consult | $167 |  | $207 |
| 77470 |  | special treatment procedure | $158 | 5623 |  | special treatment procedure | $506 |  | $411 |
|  |  |  |  |  |  |  |  |  |  |
| Total planning | | |  |  |  |  |  |  | $2,618 |

**EBRT treatment total**

|  |  | **Description** | **Reimbursement** | **APC** |  | **Description** | **Reimbursement** |  | **Total Reim** |
| --- | --- | --- | --- | --- | --- | --- | --- | --- | --- |
| 77280 |  | Simple simulation | $276 | 5612 |  | Simple simulation | $167 |  | $443 |
| 77336 |  | Weekly continuing medical physics | $80 | 5611 |  | Weekly continuing medical physics | $107 |  | $134 |
| 77402 |  | Radiation - simple | $31 | 5621 |  | Radiation therapy | $110 |  | $86 |
| 99219 |  | Initial observation care | $137 |  |  |  |  |  | $137 |
| 99225 |  | Subsequent observation care | $73 |  |  |  |  |  | $73 |
| Total treatment | | |  |  |  |  |  |  | $873 |
|  |  |  |  |  |  |  |  |  |  |
| EBRT X 6 treatments + 6 simulations each time | | |  |  |  |  |  |  | $9,237 |
|  |  |  |  |  |  |  |  |  |  |
| Total lumpectomy plus planning plus treatment | | |  |  |  |  |  |  | $13,832 |

**Life expectancies**

| Combining Means and Standard Deviations for **Locoregional recurrence** cancer survival | | | | | | | |  |
| --- | --- | --- | --- | --- | --- | --- | --- | --- |
| Survival in months |  |  |  |  |  |  |  |  |
| Mean and 95% CI calculated using QuickCalc | | |  |  |  |  |  |  |
| Variable |  | **Mean** |  | **SD** |  | **Sample size** |  | **Source** |
| Local recurrence from time of initial treatment |  | 60 |  | 6 |  | 528 |  | Touboul E, et al. Local recurrences and distant metastases after breast conserving surgery and radiation therapy for early breast cancer. Int Jrl Rad Oncol 1999. 43(1):25-38 |
| Survival time after recurrence |  | 51.75 |  | 28.75 |  | 165 |  | Kim H, et al. Prognostic factors for survivals from first relapse in breast cancer patients: analysis of deceased patients. Rad Oncol Jr. 2013. 31(4): 222-227. |
|  |  |  |  | 95% CI |  |  |  |  |
| Combined mean and 95% CIs in mths |  | 111.75 |  | 109.13 to 114.37 |  |  |  |  |
| Conversion to years |  | 9.31 |  | 9.09 to 9.53 |  |  |  |  |

| Combining Means and Standard Deviations for **Metastatic cancer** survival | | | | |  |  |  |  |
| --- | --- | --- | --- | --- | --- | --- | --- | --- |
| Survival in months |  |  |  |  |  |  |  |  |
| Mean and 95% CI calculated using QuickCalc | |  |  |  |  |  |  |  |
| Variable |  | **Mean** |  | **SD** |  | **Sample size** |  | Source |
| Metastatic recurrence from time of initial treatment |  | 49.6 |  | 5.4 |  | 528 |  | Touboul E, et al. Local recurrences and distant metastases after breast conserving surgery and radiation therapy for early breast cancer. Int Jrl Rad Oncol 1999. 43(1):25-38 |
| Survival time after recurrence |  | 18 |  | 25.97 |  | 261 |  | Chang J et al. Survival of patients with metastatic breast carcinoma. Cancer. 2003;97:545-53. |
|  |  |  |  | 95% CI |  |  |  |  |
| Combined mean and 95% CIs in mths |  | 67.5 |  | 65.19 to 69.81 |  |  |  |  |
|  |  |  |  |  |  |  |  |  |
| Conversion to years |  | 5.625 |  | 5.43 to 5.82 |  |  |  |  |

**Life expectancies based on exposures to radiation**

|  |  |  | **Comp rate** | **Comp rate** | **Comp as a percent** | **Comp as a percent** |  |  |  |  |
| --- | --- | --- | --- | --- | --- | --- | --- | --- | --- | --- |
| **Complication** | **Ther.** | **Assumed radiation exposure** | **IORT** | **EBRT** | **all comps IORT** | **all comps EBRT** | **LE (years)** | **Blended life expect EBRT** | **Blended LE IORT** | **Source** |
| Major coronary event [MCE] (MI, death, coronary revasc) | EBRT | 5 GY; assumes 35% relative increase in rate of MCE; initial rate of with no pre-exist factors 4.5%. 35% increase from 4.5% is 6.1%; absolute increase of 1.6% |  | 1.60% |  | 33.90% | 4.40 | 1.49 |  | Crimmins EM et al. Life with and without heart disease among women and men. Jrl Women Aging. 2008. 20(1-2):5-19. Crimmins EM et al. Life with and without disease: women experience more of both. 2002. 14(102):47-59. |
| Major coronary event [MCE] (MI, death, coronary revasc) | IORT | 1.25 GY; assumes 5% relative increase in rate of MCE; initial rate of with no pre-exist factors 4.5%. 5% increase from 4.5% is 4.725%; absolute increase of 0.225% | 0.23% |  | 73.77% |  | 4.40 |  | 3.25 | Crimmins EM et al. Life with and without heart disease among women and men. Jrl Women Aging. 2008. 20(1-2):5-19. Crimmins EM et al. Life with and without disease: women experience more of both. 2002. 14(102):47-59. |
| Lung cancer | EBRT | 3.4 GY for ipsilateral lung |  | 2.90% |  | 61.44% | 1.33 | 0.82 |  | Hurria A et al. Management of lung cancer in older adults. CA Cancer J Clin. 2003. 53:325-341 |
| Lung cancer | IORT | 0.03 GY for ipsilateral lung | 0.02% |  | 6.56% |  | 1.33 |  | 0.09 | Hurria A et al. Management of lung cancer in older adults. CA Cancer J Clin. 2003. 53:325-341 |
| Contralateral breast cancer | EBRT |  |  | 0.22% |  | 4.66% | 4.31 | 0.20 |  | Kim H, et el. Prognostic factors for survivals for first relapse in breast cancer patients: analysis  of deceased patients. LRR to death 4.31 +/- 2.4 years |
| Contralateral breast cancer | IORT |  | 0.06% |  | 19.67% |  | 4.31 |  | 0.85 | Kim H, et el. Prognostic factors for survivals for first relapse in breast cancer patients: analysis  of deceased patients. LRR to death 4.31 +/- 2.4 years |
| Total percentages |  |  | 0.31% | 4.72% |  |  |  | 2.51 | 4.18 |  |

**Costs – first year, annual, and end of life**

| First year |  |  | **Comp rate** | **Comp rate** | **Comp as a %** | **Comp as a %** | **Cost to treat** | **Blended cost IORT** | **Blended cost EBRT** |  |
| --- | --- | --- | --- | --- | --- | --- | --- | --- | --- | --- |
| **Complication** | **Ther** | **Assumed radiation exposure** | **IORT** | **EBRT** | **all comps IORT** | **all comps EBRT** | **event** | **to treat initial event** | **to treat initial event** | **Source** |
| Major coronary event [MCE] (MI, death, coronary revascularization) | EBRT | 5 GY; assumes 35% relative increase in rate of MCE; initial rate of with no pre-exist factors 4.5%. 35% increase from 4.5% is 6.1%; absolute increase of 1.6% |  | 1.60% |  | 33.90% | $27,771 |  | $9,414 | Darby SC et al. Risk of ischemic heart disease in women after radiotherapy for breast cancer. NEJM. 2013;368-987-98. |
| Major coronary event [MCE] (MI, death, coronary revascularization) | IORT | 1.25 GY; assumes 5% relative increase in rate of MCE; initial rate of with no pre-exist factors 4.5%. 5% increase from 4.5% is 4.725%; absolute increase of 0.225% | 0.23% |  | 73.77% |  | $27,771 | $20,487 |  | Darby SC et al. Risk of ischemic heart disease in women after radiotherapy for breast cancer. NEJM. 2013;368-987-98. |
| Lung cancer | EBRT | 3.4 GY for ipsilateral lung |  | 2.90% |  | 61.44% | $51,268 |  | $31,499.41 | Aziz MH et al. Can the risk of secondary cancer induction after breast conserving therapy be reduced using IORT with low-energy X-rays? Radiation Oncol 2011. 6:174. Ng J, et al. Predicting the risk of secondary lung malignancies associated with whole-breast radiation therapy. Int Jr. Rad Oncol 2011. |
| Lung cancer | IORT | 0.03 GY for ipsilateral lung | 0.02% |  | 6.56% |  | $51,268 | $3,361.84 |  | Aziz MH et al. Can the risk of secondary cancer induction after breast conserving therapy be reduced using IORT with low-energy X-rays? Radiation Oncol 2011. 6:174 |
| Contralateral breast cancer | EBRT |  |  | 0.22% |  | 4.66% | $11,220 |  | $522.97 | Aziz MH et al. Can the risk of secondary cancer induction after breast conserving therapy be reduced using IORT with low-energy X-rays? Radiation Oncol 2011. 6:174 |
| Contralateral breast cancer | IORT |  | 0.06% |  | 19.67% |  | $10,248 | $2,016 |  | Aziz MH et al. Can the risk of secondary cancer induction after breast conserving therapy be reduced using IORT with low-energy X-rays? Radiation Oncol 2011. 6:174 |
| Total percentages |  |  | 0.31% | 4.72% |  |  |  | $25,865 | $41,436 |  |

| **Costs to treat annual** |  |  | **Comp rate** | **Comp rate** | **Comp as a %** | **Comp as a %** | **Cost to treat** | **Blended cost IORT** | **Blended cost EBRT** |
| --- | --- | --- | --- | --- | --- | --- | --- | --- | --- |
|  |  |  | **IORT** | **EBRT** | **all comps IORT** | **all comps EBRT** | **event** | **to treat initial event** | **to treat initial event** |
| **Complication** | **Therapy** | **Assumed radiation exposure** |  |  |  |  |  |  |  |
| Major coronary event [MCE] (MI, death, coronary revascularization) | EBRT | 5 GY; assumes 35% relative increase in rate of MCE; initial rate of with no pre-exist factors 4.5%. 35% increase from 4.5% is 6.1%; absolute increase of 1.6% |  | 1.60% |  | 33.90% | $23,512 |  | $7,970 |
| Major coronary event [MCE] (MI, death, coronary revascularization) | IORT | 1.25 GY; assumes 5% relative increase in rate of MCE; initial rate of with no pre-exist factors 4.5%. 5% increase from 4.5% is 4.725%; absolute increase of 0.225% | 0.23% |  | 73.77% |  | $23,512 | $17,345 |  |
| Lung cancer | EBRT | 3.4 GY for ipsilateral lung |  | 2.90% |  | 61.44% | $5,685 |  | $3,492.90 |
| Lung cancer | IORT | 0.03 GY for ipsilateral lung | 0.02% |  | 6.56% |  | $5,685 | $372.79 |  |
| Contralateral breast cancer | EBRT |  |  | 0.22% |  | 4.66% | $1,768 |  | $82.41 |
| Contralateral breast cancer | IORT |  | 0.06% |  | 19.67% |  | $1,768 | $348 |  |
| Total percentages |  |  | 0.31% | 4.72% |  |  |  | $18,066 | $11,545 |

| **End of Life Care** |  |  | **Comp rate** | **Comp rate** | **Comp as a %** | **Comp as a %** | **Cost to treat** | **Blended cost IORT** | **Blended cost EBRT** |
| --- | --- | --- | --- | --- | --- | --- | --- | --- | --- |
| **Complication** | **Therapy** | **Assumed radiation exposure** | **IORT** | **EBRT** | **all comps IORT** | **all comps EBRT** | **event** | **to treat initial event** | **to treat initial event** |
| Major coronary event [MCE] (MI, death, coronary revascularization) | EBRT | 5 GY; assumes 35% relative increase in rate of MCE; initial rate of with no pre-exist factors 4.5%. 35% increase from 4.5% is 6.1%; absolute increase of 1.6% |  | 1.60% |  | 33.90% | $57,600 |  | $19,525 |
| Major coronary event [MCE] (MI, death, coronary revascularization) | IORT | 1.25 GY; assumes 5% relative increase in rate of MCE; initial rate of with no pre-exist factors 4.5%. 5% increase from 4.5% is 4.725%; absolute increase of 0.225% | 0.23% |  | 73.77% |  | $57,600 | $42,492 |  |
| Lung cancer | EBRT | 3.4 GY for ipsilateral lung |  | 2.90% |  | 61.44% | $74,815 |  | $45,966.84 |
| Lung cancer | IORT | 0.03 GY for ipsilateral lung | 0.02% |  | 6.56% |  | $74,815 | $4,905.90 |  |
| Contralateral breast cancer | EBRT |  |  | 0.22% |  | 4.66% | $38,879 |  | $1,812.16 |
| Contralateral breast cancer | IORT |  | 0.06% |  | 19.67% |  | $38,879 | $7,648 |  |
| Total percentages |  |  | 0.31% | 4.72% |  |  |  | $55,046 | $67,304 |

**Quality of life - initial**

| **QOL initial** |  |  | **Comp rate** | **Comp rate** | **Comp as a percent** | **Comp as a percent** |  |  |  |  |
| --- | --- | --- | --- | --- | --- | --- | --- | --- | --- | --- |
| **Complication** | **Ther** | **Assumed radiation exposure** | **IORT** | **EBRT** | **all comps IORT** | **all comps EBRT** | **Quality Life initial** | **Blended QOL initial EBRT** | **Blended QOL initial IORT** | **Source** |
| Major coronary event [MCE] (MI, death, coronary revascularization) | EBRT | 5 GY; assumes 35% relative increase in rate of MCE; initial rate of with no pre-exist factors 4.5%. 35% increase from 4.5% is 6.1%; absolute increase of 1.6% |  | 1.60% |  | 33.90% | 0.83 | 0.28 |  | Lewis EF, et al. Impact of cardiovascular events on change in quality of life and utilities in patients after MI. JACC Heart Failure. 2014. 2(2): 159-165. Table 1 page 162 - baseline utility US |
| Major coronary event [MCE] (MI, death, coronary revascularization) | IORT | 1.25 GY; assumes 5% relative increase in rate of MCE; initial rate of with no pre-exist factors 4.5%. 5% increase from 4.5% is 4.725%; absolute increase of 0.225% | 0.23% |  | 73.77% |  | 0.83 |  | 0.61 | Lewis EF, et al. Impact of cardiovascular events on change in quality of life and utilities in patients after MI. JACC Heart Failure. 2014. 2(2): 159-165. Table 1 page 162 - baseline utility US |
| Lung cancer | EBRT | 3.4 GY for ipsilateral lung |  | 2.90% |  | 61.44% | 0.63 | 0.39 |  | Lairson DR, et al. Cost-utility analysis of treatments for advanced non-small cell lung cancer. Am Jrl Pharm Benefits. 2015. 7(6):271-279. |
| Lung cancer | IORT | 0.03 GY for ipsilateral lung | 0.02% |  | 6.56% |  | 0.63 |  | 0.04 | Lairson DR, et al. Cost-utility analysis of treatments for advanced non-small cell lung cancer. Am Jrl Pharm Benefits. 2015. 7(6):271-279. |
| Contralateral breast cancer | EBRT |  |  | 0.22% |  | 4.66% | 0.70 | 0.03 |  | Hillner BE, Smith TJ. Efficacy and cost effectiveness of adjuvant chemotherapy in women with node-negative breast cancer. NEJM. 1991. 324:160-168. Table 1 page 162 |
| Contralateral breast cancer | IORT |  | 0.06% |  | 19.67% |  | 0.70 |  | 0.14 | Hillner BE, Smith TJ. Efficacy and cost effectiveness of adjuvant chemotherapy in women with node-negative breast cancer. NEJM. 1991. 324:160-168. Table 1 page 162 |
| Total percentages |  |  | 0.31% | 4.72% |  |  |  | 0.70 | 0.79 |  |

**Quality of life stable disease**

| **QOL stable disease** |  |  | **Comp rate** | **Comp rate** | **Comp as a percent** | **Comp as a percent** |  |  |  |  |  |
| --- | --- | --- | --- | --- | --- | --- | --- | --- | --- | --- | --- |
| **Complication** | **Ther** | **Assumed radiation exposure** | **IORT** | **EBRT** | **all comps IORT** | **all comps EBRT** | **QOL stable disease** | **Blended QOL stable EBRT** | **Blended QOL stable IORT** | **Source:** |  |
| Major coronary event [MCE] (MI, death, coronary revascularization) | EBRT | 5 GY; assumes 35% relative increase in rate of MCE; initial rate of with no pre-exist factors 4.5%. 35% increase from 4.5% is 6.1%; absolute increase of 1.6% |  | 1.60% |  | 33.90% | 0.76 | 0.26 |  | Lewis EF, et al. Impact of cardiovascular events on change in quality of life and utilities in patients after MI. JACC Heart Failure. 2014. 2(2): 159-165. Based on table 3, page 163 - mean trajectory change of -0.07 |  |
| Major coronary event [MCE] (MI, death, coronary revascularization) | IORT | 1.25 GY; assumes 5% relative increase in rate of MCE; initial rate of with no pre-exist factors 4.5%. 5% increase from 4.5% is 4.725%; absolute increase of 0.225% | 0.23% |  | 73.77% |  | 0.76 |  | 0.56 | Lewis EF, et al. Impact of cardiovascular events on change in quality of life and utilities in patients after MI. JACC Heart Failure. 2014. 2(2): 159-165. Based on table 3, page 163 - mean trajectory change of -0.07 |  |
| Lung cancer | EBRT | 3.4 GY for ipsilateral lung |  | 2.90% |  | 61.44% | 0.71 | 0.44 |  | Doyle S et al. Health state utility scores in advanced non-small cell lung cancer. Lung Cancer. 2008. 62:374-380. |  |
| Lung cancer | IORT | 0.03 GY for ipsilateral lung | 0.02% |  | 6.56% |  | 0.71 |  | 0.05 | Doyle S et al. Health state utility scores in advanced non-small cell lung cancer. Lung Cancer. 2008. 62:374-380. |  |
| Contralateral breast cancer | EBRT |  |  | 0.22% |  | 4.66% | 0.85 | 0.04 |  | Hillner BE, Smith TJ. Efficacy and cost effectiveness of adjuvant chemotherapy in women with node-negative breast cancer. NEJM. 1991. 324:160-168. Table 1 page 162 |  |
| Contralateral breast cancer | IORT |  | 0.06% |  | 19.67% |  | 0.85 |  | 0.17 | Hillner BE, Smith TJ. Efficacy and cost effectiveness of adjuvant chemotherapy in women with node-negative breast cancer. NEJM. 1991. 324:160-168. Table 1 page 162 |  |
| Total percentages |  |  | 0.31% | 4.72% |  |  |  | 0.73 | 0.77 |  | |

**Estimated Time to event after exposure to radiation from treating breast cancer**

|  |  |  | **Comp rate** | **Comp rate** | **Comp as a percent** | **Comp as a percent** |  |  |  |  |
| --- | --- | --- | --- | --- | --- | --- | --- | --- | --- | --- |
| **Complication** | **Therapy** | **Assumed radiation exposure** | **IORT** | **EBRT** | **all comps IORT** | **all comps EBRT** | **Time to first event (yrs)** | **Blended time yrs EBRT** | **Blended time yrs IORT** | **Source** |
| Major coronary event [MCE] (MI, death, coronary revascularization) | EBRT | 5 GY; assumes 35% relative increase in rate of MCE; initial rate of with no pre-exist factors 4.5%. 35% increase from 4.5% is 6.1%; absolute increase of 1.6% |  | 1.60% |  | 33.90% | 12.32 | 4.18 |  | Darby SC et al. Risk of ischemic heart disease in women after radiotherapy for breast cancer. NEJM 2013. 368(11): 987-998. Supplementary Table 2S |
| Major coronary event [MCE] (MI, death, coronary revascularization) | IORT | 1.25 GY; assumes 5% relative increase in rate of MCE; initial rate of with no pre-exist factors 4.5%. 5% increase from 4.5% is 4.725%; absolute increase of 0.225% | 0.23% |  | 73.77% |  | 12.32 |  | 9.09 | Darby SC et al. Risk of ischemic heart disease in women after radiotherapy for breast cancer. NEJM 2013. 368(11): 987-998. Supplementary Table 2S |
| Lung cancer | EBRT | 3.4 GY for ipsilateral lung |  | 2.90% |  | 61.44% | 10.39 | 6.38 |  | de Gonzalez, AB. Second solid tumors after radiotherapy for breast cancer in SEER cancer registries. BJC 2010. 102:220-226. Table 3 |
| Lung cancer | IORT | 0.03 GY for ipsilateral lung | 0.02% |  | 6.56% |  | 10.39 |  | 0.68 | de Gonzalez, AB. Second solid tumors after radiotherapy for breast cancer in SEER cancer registries. BJC 2010. 102:220-226. Table 3 |
| Contralateral breast cancer | EBRT |  |  | 0.22% |  | 4.66% | 9.73 | 0.45 |  | de Gonzalez, AB. Second solid tumors after radiotherapy for breast cancer in SEER cancer registries. BJC 2010. 102:220-226. Table 4 |
| Contralateral breast cancer | IORT |  | 0.06% |  | 19.67% |  | 9.73 |  | 1.91 | de Gonzalez, AB. Second solid tumors after radiotherapy for breast cancer in SEER cancer registries. BJC 2010. 102:220-226. Table 4 |
| Total percentages |  |  | 0.31% | 4.72% |  |  |  | 11.01 | 11.68 |  |
